# Modeling migration patterns in the USA under sea level rise

**DOI:** 10.1371/journal.pone.0227436

**Published:** 2020-01-22

**Authors:** Caleb Robinson, Bistra Dilkina, Juan Moreno-Cruz

**Affiliations:** 1 School of Computational Science and Engineering, Georgia Institute of Technology, Atlanta, Georgia, United States of America; 2 Viterbi School of Engineering, University of Southern California, Los Angeles, California, United States of America; 3 School of Environment, Enterprise and Development, University of Waterloo, Waterloo, Ontario, Canada; University of Alabama, UNITED STATES

## Abstract

Sea level rise in the United States will lead to large scale migration in the future. We propose a framework to examine future climate migration patterns using models of human migration. Our framework requires that we distinguish between historical versus climate driven migration and recognizes how the impacts of climate change can extend beyond the affected area. We apply our framework to simulate how migration, driven by sea level rise, differs from baseline migration patterns. Specifically, we couple a sea level rise model with a data-driven model of human migration and future population projections, creating a generalized joint model of climate driven migration that can be used to simulate population distributions under potential future sea level rise scenarios. The results of our case study suggest that the effects of sea level rise are pervasive, expanding beyond coastal areas via increased migration, and disproportionately affecting some areas of the United States.

## Introduction

Climate change is already affecting millions of people around the world [1]. Human migration is a natural response to these climate change pressures, and is one of many adaptation measures that people will take in response to climate change [2–5]. Understating how human migration will be affected by climate change is therefore a critical input in the decision making process of many governments and organizations. In particular, it is important to understand how climate change driven migration will differ from “business as usual” forms and motivations humans have to migrate. Yet, an empirical assessment of the process remains elusive. In this paper, we propose a framework that recognizes the imperfections of any given assessment regarding migration, but allows us to think critically about the possible ways climate change can alter migration patterns.

In particular, our framework assesses the broader impacts of climate change on population, by explicitly considering the effects on migration on populations directly affected by climate change and *indirectly affected* by the change in migration patterns induced by climate change. The framework we propose here is not intended to explain individual decisions, but to characterize aggregate patterns that can nonetheless help prepare policy responses by local communities and governments.

Accounting for those indirectly affected people is one of the main contributions of our framework; these are people that live in locations that experience increased population pressures due to heightened inflows of climate migrants. These indirect effects will cause accelerated changes for inland areas, particularly urban areas, that will observe much higher levels of incoming migrants than they would have without climate impacts. These changes can in turn take the form of tighter labor markets [6] and increased housing prices [7], with broader effects on income inequality in the coastal areas [8]. Of course, migration to other cities can also have positive impacts; new migrants can improve productivity as they bring with them human capital accumulated elsewhere [5]. Thus, explicitly accounting for *indirect effects*, even under large uncertainties, is an important part of quantifying the effects of climate change.

Broadly speaking, migration processes can be characterized by three components: sources, destinations, and flows between them. Climate change will affect each of these components in different ways. For example, increased climate burden on agricultural regions can increase migrants to move to more urban spaces or to move to different towns, provinces or even different countries. Climate change can also induce conflict, thus increasing the number of refugees. Climate change can also affect destinations, for example by making cities less livable due to urban heat island effects [9] or due to increased burden on services such as water and electricity [10]. By affecting both the origin and destination, climate change also affects the flow of migrants. Our proposed framework *explicitly separates* climate change driven and “business as usual” migrations and therefore allows future modeling improvements in either of these processes to be easily incorporated.

We introduce and discuss our framework in the context of sea level rise impacts on human migration. Sea level rise (SLR) will affect millions of people living in coastal areas. Different studies have highlighted likely scenarios of sea level rise by 2100, varying in their projections of severity. According to the IPCC Fifth Assessment Report, in the “worst-case” Representative Concentration Pathways (RCP) scenario, RCP 8.5, where greenhouse gas emissions continue to rise throughout the 21st century, a global mean sea level (GMSL) rise between 0.52 to 0.98 meters (m) is likely by 2100 [11]. Other estimates, using statistical instead of process based models of GMSL, project a rise in the range of 0.75 m to 1.9 m by 2100 [12]. Recent research from the National Oceanic and Atmospheric Administration (NOAA) has even suggested a 2.5 m upper bound of GMSL rise by 2100 for an ‘extreme’ SLR scenario, and a 2 m GMSL rise for a ‘high’ scenario [13].

The impacts of SLR are potentially catastrophic. About 30% of the urban land on earth was located in high-frequency flood zones in 2000, and it is projected to increase to 40% by 2030 taking urban growth and SLR into account [14]. In the United States alone, 123.3 million people, or 39% of the total population, lived in coastal counties in 2010, with a predicted 8% increase by the year 2020 [15]. By the year 2100, a projected 13.1 million people in the United States alone would be living on land that will be considered flooded with a SLR of 6 feet (1.8 m) [16].

As oceans expand and encroach into previously habitable land, affected people—climate migrants—will move towards locations further inland, looking for food and shelter in areas that are less susceptible to increased flooding or extreme weather events. In this paper, we argue that the comprehensive impacts of SLR on human populations, when considering migration, expand far beyond the coastal areas.

While discussions regarding SLR impacts on human populations are often constrained to regions *directly* experiencing SLR-driven flooding [16–18], several studies have investigated the connection to climate driven human migration. Several theoretical frameworks use qualitative case studies to motivate models that might represent the reasoning behind migration choices due to SLR, but are not grounded in statistical methods [2, 19, 20]. There are many complex interactions between demographic driven migration and climate change driven migration, and the scope and scale of the impacts of climate change on migration will be significant [3, 21]. One example of these impacts that has been studied considers the political ramifications that will come with the eventual migrants from Pacific island of Kiribati, which will most likely become completely flooded under a 3 meter SLR [22]. Another example is the projected widening demographic differentials in countries that will be especially impacted by SLR [21, 23], similar to the demographic changes seen after the 1970s droughts in Africa [24]. The foundation of both of these concerns is in people’s destination locations, therefore it is prudent to weigh the question of ‘where’ people will go equally with ‘how many’ people will be initially affected [25].

There are also few empirical studies that link climate change with human migration patterns. Feng et al. show that the negative impacts of climate change on crop-yields has driven increased emigration from Mexico to the United States [3], while Thiede and Gray examine the effects of changing climate variables on the timing of migration in Indonesia [26]. The only empirical works that examine the *effects of SLR on human migration* do so by coupling population projections with SLR models and migration models to estimate how population distributions might change in future scenarios [16, 27, 28]. In the US, small area population projections for the year 2100 have been combined with spatially explicit estimates of SLR [16] and an unobserved component regression model to estimate the destinations of populations that could be forced to migrate through coastal flooding. In [27], approximately 56% of counties in the US are found to be affected by larger migrant influxes under 1.8 m of SLR. Similarly, in Bangladesh, gridded population projections have been combined with a connectivity aware bathtub type model of SLR and the radiation model of human migration to estimate how population distributions may change [28]. This coupled model has minimal data requirements, forecasts large quantities of immigration to the division of Dhaka in Bangladesh, and highlights the broader potential impacts of these migrants including an increased demand for housing, food, and jobs.

These empirical studies make the critical simplifying assumption that climate driven migration will follow the same patterns as historic migration. In fact, even though Hauer [27] highlights that “climate migrants resulting from press stressors will probably constitute ‘enhanced’, or extra, normal out-migration”, in the paper he assumes that migrations will happen only between locations for which there are historically observed migrations. However, human migration is a function of push and pull factors, and increased climate stress will affect both [19]. Our framework, by incorporating separate models of migration choices for climate change driven versus “business-as-usual” migration, recognizes that the patterns of climate migrants will not necessarily follow patterns observed in historical migration data. In our application, this distinction proves to be important.

To apply our framework to our case study on the impacts of SLR, we couple models of SLR with dynamic models of human migration to produce a more comprehensive picture of changing population distributions. We implement our framework with spatial estimates of SLR from the NOAA’s Digital Coast dataset [29], small area population projections [16], and a recent machine learning (ML) method for modeling human migration [30]. We model migrations made from *flooded* areas and from *unflooded* areas separately by fitting one ML migration model using “business-as-usual” migration data and one climate change driven model with migration data following Hurricanes Katrina and Rita. As more sophisticated high-resolution population projections are developed, human migration models are improved, and SLR projections are refined, the precision of our results will also improve.

## Modeling framework

### Conceptual challenges in coupling human migration models

Traditional strategies for modeling human migration do not lend themselves well to describing climate change driven migration. Current state-of-the-art models of human migration include the family of radiation models [31, 32], gravity models [33, 34], and machine learning models [30]. The problems are as follows:

First, human migration induced by climate change might not follow historic migration patterns. In fact, using one year of county-to-county migration data from the IRS U.S. migration data sets, Simini et al. [31] showed that a fixed proportion (3%) of the population of a U.S. county will migrate under normal circumstances. This will not hold under SLR, for example, as the entire population in flooded areas will have to move or adapt in other ways. Importantly, in addition to direct inundation due to SLR, climate migrants will be forced to move as climate change effects become more pronounced, directly through the exposure to “high-magnitude events” such as large scale flooding from hurricanes, or indirectly through the “cumulative contribution of ongoing localized events across regions” [35]. The dynamics of these environmentally induced migrations will not necessarily follow those of previously observed migrations. We expect that as social scientists gather more data and knowledge, more refined and informed models of human migration will emerge.

Second, the spatial resolution of each model must be carefully considered. Climate migrants will not necessarily move large distances as they adapt to changing conditions in inundated areas. Indeed, per the US IRS migration data [36], most migrations are made to nearby locations. This phenomenon can be seen in the migrations following Hurricane Katrina in the US, for example, where a majority of destinations were within Louisiana [37]. Hence, it is important to be able to model such nearby migrations, including migrating from the climate affected part to the unaffected part within the same zone, as well as to exclude migrations to areas that are uninhabitable (e.g. inundated by SLR). To do finer scale modeling, the scale of population projections also matters. Population projections are an important input in modeling human migration, and integrating model results over various future population scenarios is crucial for determining uncertainty. However, most existing long term population projections are only done at country or region level scales, e.g. the variety of population projections defined in the Shared Socioeconomic Pathways [38], and can not be appropriately applied to the finer-scale resolutions at which SLR and migration models will operate.

Third, migration models and population projections are inherently coupled through a feedback loop whereby the population projections at a given time should account for the cumulative effects of climate change driven migration before then. For example, SLR will not happen instantaneously. The SLR influenced population distribution of a location will diverge from that of a “business as usual” scenario as the indirect effects of SLR compound; as more climate migrants settle inland, they will change the migrations patterns of future migrants and so on. However, current models used for population projections at best account for the *direct* effects of SLR where projected populations are made with respect to potentially flooded land [16], however the *indirect* effects must also be considered.

### General modeling framework

**Fig 1:**
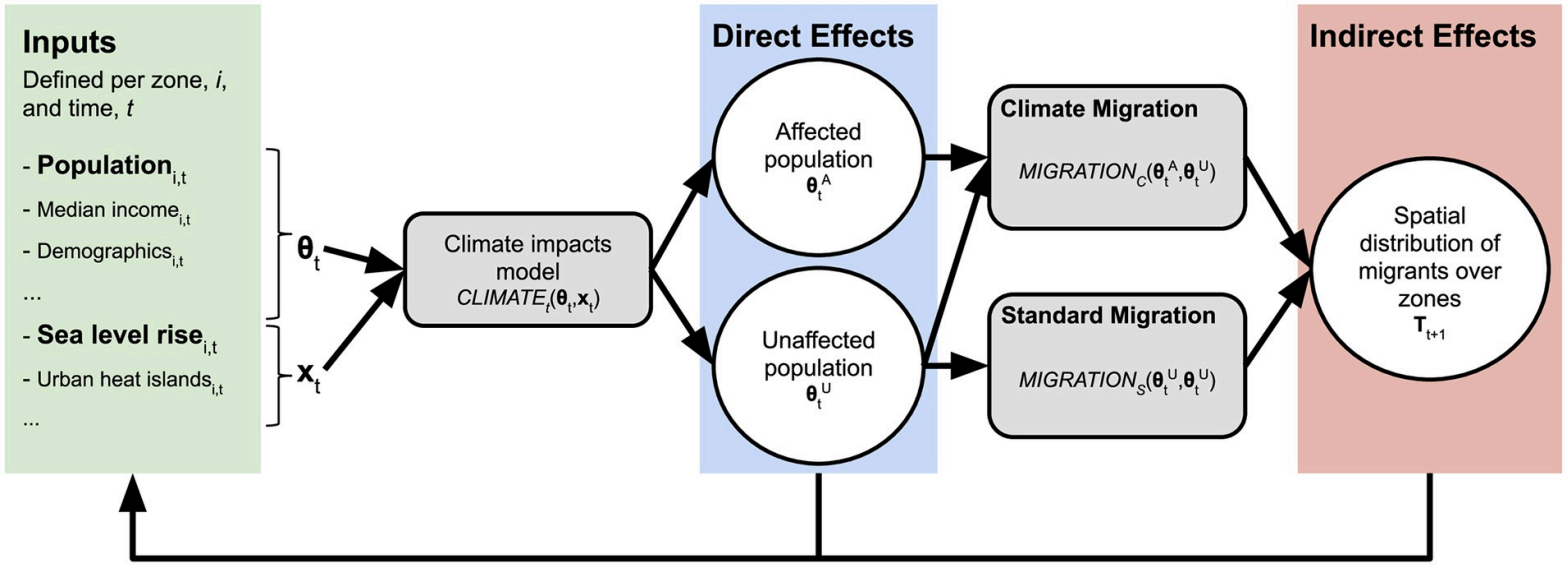
Joint climate change impact and human migration modeling process. The joint model takes a list of spatial zones ***θ*** and climate change features **x**^*t*^ as input, and outputs a *migration matrix* ***T***^*t*^, where an entry $T_{ij}^{t}$ represents the number of migrants that travel from zone *i* to *j* at time *t*.

For a given time, *t*, we will consider climate change features, **x**^*t*^, and a list of *n* spatial zones, $\theta^{t}=\left[\theta_{1}^{t},\ldots ,\theta_{n}^{t}\right]$, which includes a spatial definition and features associated with each zone. Using this information, we want to compute a *migration matrix* ***T***^*t*^, where an entry $T_{ij}^{t}$ represents the number of migrants that travel from zone *i* to *j* at time *t* under a given climate impact model. We propose a general modeling framework for handling this problem which consists of two modules, shown in Fig 1: a CLIMATE module and a MIGRATION module.

**CLIMATE module**. This module uses a “climate impacts model”, *CLIMATE*(***θ***, **x**) to partition each input zone into two new zones: the affected portion and the unaffected portion. Using the best available data, this module should also divide the *features* from the original zone (*θ*_*i*_) between the affected-portion zone ($\theta_{i}^{A}$) and the unaffected-portion zone ($\theta_{i}^{U}$). For example, if we have high-resolution spatial population data, then we can split population between the two partitions based on the spatial extent of flooding given a SLR model.

**MIGRATION module**. This module calculates ***T*** using the two sets of zones from the CLIMATE module. Specifically, this module uses two migration models: 1.) a model for migrations from affected zones ***θ***^*A*^ to unaffected zones ***θ***^*U*^ with the function *MIGRATION*_*C*_(***θ***^*A*^, ***θ***^*U*^) = **T**′, where migration is a forced process driven by climate change; and 2.) a model of migrations from unaffected zones to unaffected zones with the function *MIGRATION*_*S*_(***θ***^*U*^, ***θ***^*U*^) = **T**″, where migration happens as usual. Finally, this module should aggregate migrant flows from the two migration functions, **T** = **T**′ + **T**″.

By separating CLIMATE driven migration from “business as usual” migrations, our framework forces these dynamics to be considered independently, explicitly bringing up the issue from the first conceptual challenge mentioned in the previous section. Implementations of our framework can use different models for these dynamics if available, or, if such models are not available, fall back to using a simpler model where the simplifying assumption is clear. Our framework also addresses the second conceptual challenge by restricting destinations to only zones marked as unaffected, and allowing the affected population of a zone to choose the unaffected part as its destination. Furthermore, by separating the functionality of the CLIMATE module from that of the MIGRATION module, the framework allows ablation studies to measure how much results depend on the specific behaviors of each. The third conceptual challenge revolves around how CLIMATE and MIGRATION are both temporal processes that form a feedback loop (e.g. SLR will affect migration decisions, which will in turn affect how many people are affected by further SLR, etc.). It is captured in Fig 1 by including a conceptual link from the estimated migration matrix back into the inputs such as population projections at time *t* + 1.

Finally, our framework provides a methodology by which to calculate the *direct* and *indirect* effects of the climate change impacts model. The *direct* effects are simply the areas that are explicitly affected by the climate impacts model. The *indirect* effects, however, are a function of the changing migration patterns. Formally, a zone is marked as indirectly affected if the difference between the number of incoming migrants in the CLIMATE scenario and the baseline scenario is greater than a percentage *d*% of the population of that zone. By varying *d* we can see different intensities of indirect effects.

### Our framework instantiated with SLR-based climate impact model and ML-based migration models

We implement our proposed framework considering SLR as the driver of the climate impacts module. An implementation of our framework requires us to define the manner in which a CLIMATE function splits features associated with the zones that are affected by climate change, and two MIGRATION functions. All three of these steps are discussed in the next two sections. We use SLR spatial estimates based on the NOAA’s Digital Coast dataset [29], spatial population projections following the methodology in [16], and a recent machine learning (ML) approach for modeling human migration [30]. All the data sources we use are listed in the S2 Table.

#### SLR-based climate impacts model

First, we use the spatial estimates of SLR from the Digital Coast dataset [29] to determine which census block groups will be flooded under a given estimate of SLR, *x*. The Digital Coast spatial estimates take tidal variability, hydroconnectivity, probable flooding, and federal leveed areas into account to present a plausible estimate of inundated areas at different *estimates* of SLR, however it is not directly linked to climate projections and does not use more complicated physical flood models. The Digital Coast dataset represents a more serious modeling effort than a “bathtub model” (where using elevation data one marks land under *x* meters as flooded assuming that sea level will rise uniformly across all coastlines), but can be improved as country-wide sea level rise estimates are improved. We estimate *when* different estimates of SLR considered in the Digital Coast dataset will happen using *climate projections* for two SLR scenarios: **medium** scenario, where 0.9m (3ft) of SLR is experienced by 2100, and **high** scenario, where 1.8m (6ft) of SLR is experienced by 2100. These two SLR projection scenarios are proposed in Hauer 2016 [16], based on methods from the US National Climate Assessment. The medium SLR scenario expects SLR to reach the 0.3m, 0.6m, and 0.9m thresholds in the years 2055, 2080, and 2100 respectively, while the high scenario reaches 0.3m, 0.6m, 0.9m, 1.2m, 1.5m, and 1.8m in the years 2042, 2059, 2071, 2082, 2091, and 2100 respectively.

We pair the spatial flooding estimates at each SLR estimate with population projections also following the methodology in Hauer et al., 2016 [16]. Here we create population projections for *every* Census block group in the US (*n* = 216, 330). Now, for every 0.3m increment from 0 to 1.8m (corresponding to different years in medium and high scenarios) we have population estimates and area of flooding estimates for every census block group in the US.

With this data, we define the CLIMATE module with the *SLR*(*θ*_*i*_, *x*_*t*_) function that takes a *county*, *θ*_*i*_, and estimate of SLR under either the medium or high SLR scenario, *x*_*t*_, as input. A given county corresponds to a set of census block groups. A given SLR estimate, under either scenario, corresponds to population projection for each census block group, including an estimate of the number of people *affected* by flooding in each block group. For the purposes of modeling migration, we split the county into unaffected and affected portions $\theta_{i}^{U}$ and $\theta_{i}^{A}$ respectively. Here, $\theta_{i}^{U}$ is a “new” county equivalent zone with a population equal to the sum of the unaffected populations over all the associated block groups. Similarly, $\theta_{i}^{A}$, represents the inundated portion of the original county, and will contain a population equal to the sum of affected populations in the associated block groups. We can run *SLR*(*θ*_*i*_, *x*_*t*_) for all *i* to get the ***θ***^*U*^ and ***θ***^*A*^ sets. In our case strudy, population is the only feature required for each zone by the MIGRATION module, however a similar splitting technique could be used for other block group level features if they can be reliably projected into future scenarios.

#### Human migration modeling

We model human migration between counties in the USA with a recently proposed artificial neural network (ANN) based method [30] that is fit with historic county-to-county migration data from the IRS [36]. This method is similar in functionality to traditional models of human mobility and migration, such as the radiation or gravity models [31, 33, 39]. These models all predict the probability of migration between an origin and a destination as a function of population and distance. The basic intuition behind the traditional models is that probability of migration will be larger with large origin and destination populations, however will decrease with larger distances between the origin and destination. On the other hand, our ANN modeling approach is purely data driven and models the relationship between the probability of migration as a highly parameterized nonlinear function of the features of origins and destinations.

More specifically, our ANN models estimate *P*_*ij*_, the probability that a migrant which leaves an origin county, *i*, will travel to a destination county, *j*, as a non-linear function of: origin population, *m*_*i*_, destination population, *m*_*j*_, great-circle distance between the two, *d*_*ij*_, and the “intervening opportunities” between the two, *s*_*ij*_ (this is the total population in the circle centered at *i* with radius *d*_*ij*_, not including *m*_*i*_ or *m*_*j*_). These features are the same features used by traditional radiation and gravity models, and depend solely on population and distance. While the ANN uses the same set of features, it does not have a fixed functional form and can learn complex non-linear relationships between the features and the target output (*P*_*ij*_).

To compute *T*_*ij*_, the number of migrants that travel from *i* to *j*, we need to know the number of migrants that are attempting to leave *i*. If we say that the number of migrants leaving zone *i* is of the form *g*(*m*_*i*_) = *αm*_*i*_, where *α* is some coefficient that specifies the fraction of the total population that will migrate, then *T*_*ij*_ = *g*(*m*_*i*_)*P*_*ij*_. This function *g* is called the production function. Now we can define the MIGRATION functions, *MIGRATION*_*C*_ and *MIGRATION*_*S*_, which represent the climate migrants and “business as usual” migrants respectively, by training two instances of our ANN model, and forming respective production functions *g*_*C*_(*m*_*i*_) and *g*_*S*_(*m*_*i*_) by choosing *α*_*C*_ and *α*_*S*_.

We fit the *MIGRATION*_*C*_ model by finding hurricane affected counties from the IRS migration data from 2004-2011 and 2011-2014. We did not evaluate 2010-2011 change as the reporting methodology in the IRS migration dataset changed between the 2010-2011 data and 2011-2012 data. Specifically, we search for migration data points (i.e. pairs of counties) in which the origin county was a coastal county that observed an over 100% *increase* in outgoing migrations with over 1,000 total outgoing migrations. This “filter” highlights counties that have potentially been affected by hurricanes or other natural disasters and indeed finds seven counties from 2005 that were heavily impacted by hurricanes Katrina and Rita: St Bernard, Orleans, Cameron, Plaquemines, Hancock, Jefferson, and Harrison (which matches literature estimates of the most damaged counties [37]), as well as Liberty County, GA from 2006. The only historical explanation we can find for a sudden increase in outgoing migration in Liberty County is the deactivation of a large US military division stationed within the county that year. Considering this, we fit our *MIGRATION*_*C*_ model using the data from the seven counties that were most seriously affected by hurricanes Katrina and Rita. As background, Hurricane Katrina struck the city of New Orleans and the broader Louisiana and Mississippi coastline on August 29, 2005 causing widespread flooding and wind damage. Less than a month later, on September 25, Hurricane Rita also struck the Louisiana coast, exacerbating damage in New Orleans and causing extensive damage to counties in the western portions of the state. Over 1500 people were killed and over 80% of the city of New Orleans was flooded as a result of these hurricanes. Followup studies and Census estimates showed that New Orleans only contained around half of its pre-hurricane population within a year of the storms. By training our ANN with these counties we allow the model to pick up on the dynamics of migrations after extreme flooding events.

We train an ANN, $MIGRATION_{C}\left(\theta_{i}^{A},\theta_{j}^{U}\right)=P_{ij}^{\prime }$, using all 7 × 3099 pairs of counties from the 2005-2006 IRS data that include one of the seven previously mentioned *affected* counties as an origin and an *unaffected* county as a destination. Similarly, we fit another ANN using the rest of the IRS migration data, $MIGRATION_{S}\left(\theta_{i}^{U},\theta_{j}^{U}\right)=P_{ij}^{\prime \prime }$. Due to our assumption that all people in flooded areas will have to migrate, the production function for climate migrants is given as the identity, *g*_*C*_(*m*_*i*_) = *m*_*i*_. This forces the entire population of the affected portions of counties to become migrants. For “business as usual” migrants, we use the production function, *g*_*S*_(*m*_*i*_) = 0.03*m*_*i*_, due to the observation by Simini et al. [31] that 3% of a county’s population will migrate under normal conditions each year. Given these: $T_{ij}^{\prime }=g_{C}\left(m_{i}\right)MIGRATION_{C}\left(\theta_{i}^{A},\theta_{j}^{U}\right)$, and $T_{ij}^{\prime \prime }=g_{S}\left(m_{i}\right)MIGRATION_{S}\left(\theta_{i}^{U},\theta_{j}^{U}\right)$. With these definitions we can build **T**′ and **T**″ by running the climate migration ANN and “business as usual” migration ANN for all pairs of counties.

In Section 2 of the S1 Appendix we show a similar implementation using the Extended Radiation model [32] to implement the MIGRATION functions. In Section 3 of the S1 Appendix we describe our ML model and give validation results comparing our ANN based migration model to other human migration models on the task of predicting inter-county migrations in the US.

## Results

**Fig 2:**
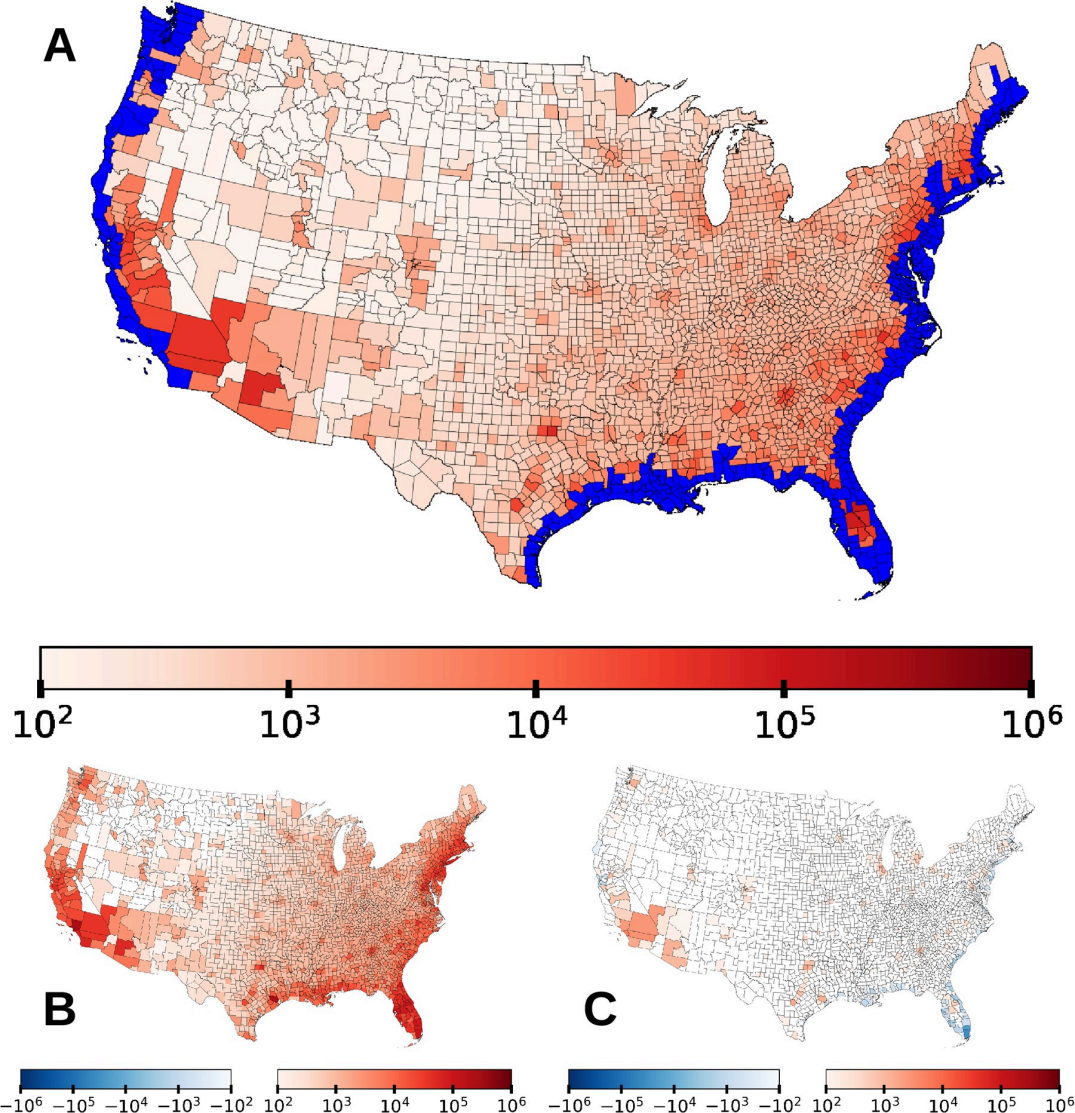
Spatial distribution of the direct and indirect effects of SLR on human migration. The **top** panel shows all counties that experience flooding under 1.8m of SLR by 2100 in blue and colors the remaining counties based on the number of additional incoming migrants per county that there are in the SLR scenario over the baseline. The **bottom left** map shows the number of additional incoming migrants per county in the SLR scenario from only flooded counties. The **bottom right** map shows the number of additional incoming migrants per county in the SLR scenario from only unflooded counties. Color gradients are implemented in a log scale.

**Fig 3:**
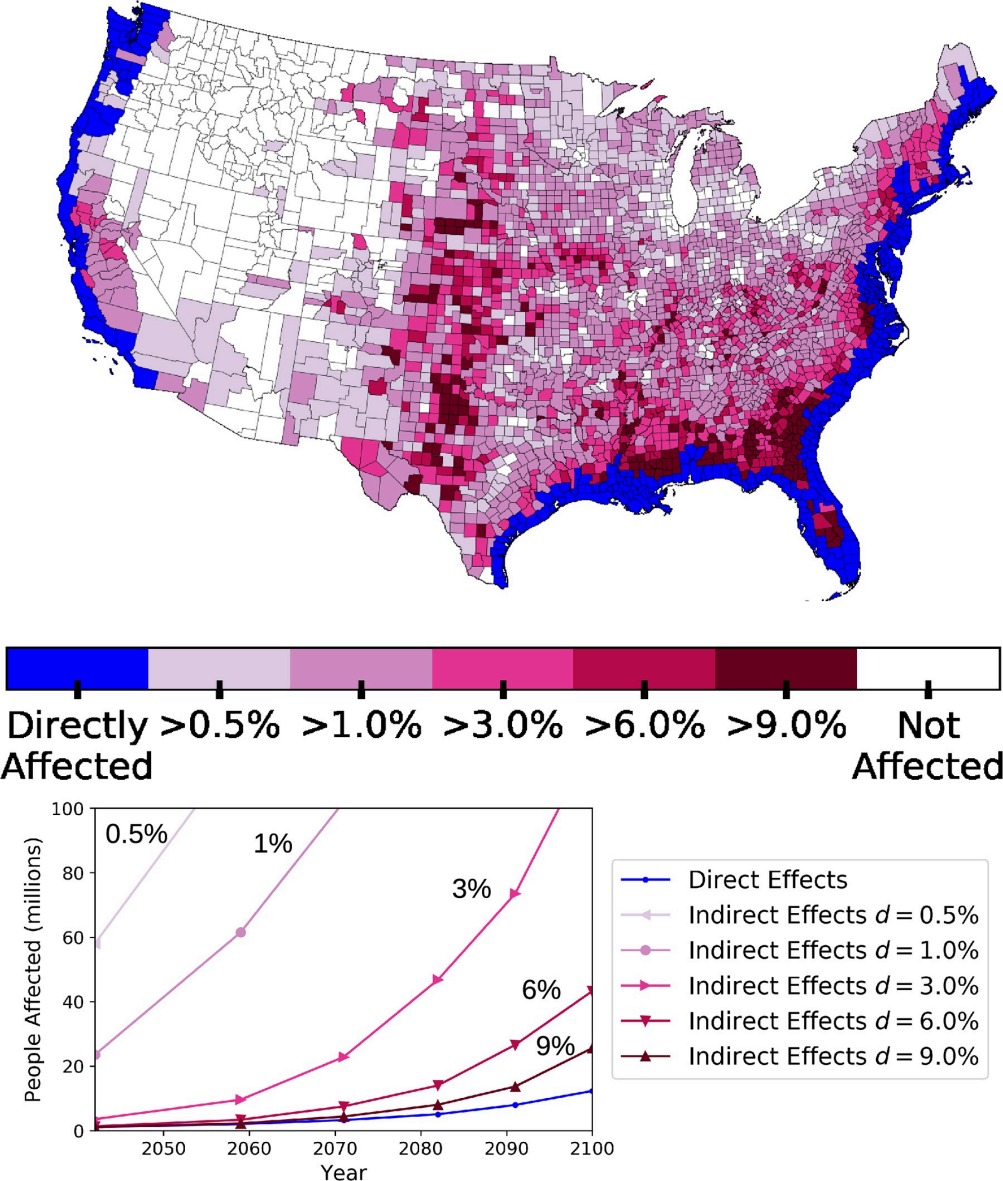
Impacts of SLR due to flooding and human migration for a range of SLR scenarios. We say that a county is indirectly affected by SLR if the difference between the number of incoming migrants to the county in the SLR scenario and the number of incoming migrants in the baseline scenario, i.e. the number of *extra* migrants in the SLR scenario, is greater than some percentage, *d*, of that county’s population. In the **top** panel we show the spatial distribution of counties that are considered indirectly affected at different threshold values of *d* for the 1.8m SLR case in the southeast portion of the United States. In the **bottom** panel we show the number of people that are directly and indirectly affected under the same threshold values of *d* for the entire United States. For both plots we show aggregate impacts for five different values of *d*: 0.5%, 1%, 3%, 6%, and 9%.

We categorize the effects of SLR into two types: *direct effects*, which are a direct consequence of SLR, and *indirect effects*, which are a consequence of changing migration patterns due to SLR. We present the spatial distribution and magnitude of these effects in Figs 2 and 3. People that live on flooded land who will have to move away are accounted for in the *direct effects* of SLR. People that live in counties that experience a larger number of incoming migrants in the flooding scenario relative to the baseline scenario with no SLR are accounted for in the *indirect effects* of SLR.

In Fig 2 we show the spatial distribution of changes in migration patterns. In the top panel, counties experiencing any SLR inundation (i.e. that are directly affected by SLR) are highlighted in blue, while the remaining counties are colored according to how many additional migrants they receive in the 1.8m SLR scenario. The bottom two panels of Fig 2 show the difference in the number of incoming migrants between the SLR scenario and the baseline scenario for incoming migrants from *unaffected* counties and *affected* counties. The top panel is the sum of these two maps, and shows this difference for incoming migrants from *all* counties. From these maps we can see that the primary destination of climate migrants are counties just inland of their origin, but climate migrants also move farther towards large cities that offer more opportunities.

In Fig 3 we show the magnitude of the *direct* and *indirect* effects as well as the spatial distribution of the indirectly affected counties. We calculate whether a county (and hence its population) has been *indirectly affected* at a level *d*% through the methodology described in the General modeling framework section. On average, in business as usual conditions, 3% of a county’s population migrates each year [31]. Thus, if *d* = 3%, we would mark a county as indirectly affected if we observe twice as much migration into that county than the average migration rate of the US. We assume that as *d* increases, the effects will be stronger as there will be a significant strain on the resources in that particular county.

The graph in the bottom panel of Fig 3 shows the direct and indirect effects of SLR in terms of number of people affected for estimated SLR in the range from 0.3m to 1.8m in 0.3m increments. The map in the top panel shows which counties in the United States are indirectly affected at different threshold values of *d*. In both plots the indirect effects are shown for five different values of *d*: 0.5% 1%, 3%, 6%, and 9%.

We can see that the *indirect* impacts of SLR grow at much faster rate than the *direct* impacts (Fig 3). In the high SLR scenario by the year 2100 there are ≈ 13 million people *directly* affected, in ≈ 50 thousand km^2^ of flooded land, however there are almost twice as many, ≈ 25 million people, *indirectly* affected at the 9% threshold due to changing migration patterns and magnitudes. This d = 9% threshold indicates that these people live in areas which will experience four times as many migrants as they would compared to a baseline scenario. Even under the moderate assumption of 0.9m SLR by 2100 there will be 24 million people that live in counties considered indirectly affected at a d = 3% threshold. Under the same threshold with a SLR of 1.8m by 2100, there will be 120 million people, over ≈1/3 of the population of US, living in counties that will see a doubling in the number of annual incoming migrants.

The map in Fig 3 shows that these *indirect* effects relative to county population will be distributed unevenly over the US. Most effects are seen in the Eastern US, where there are more vulnerable coastal populations. Of particular note are southern Mississippi and southeastern Georgia, where large groups of counties are estimated to see indirect effects in the >9% category. The Midwest is also projected to see large indirect effects, even though the magnitudes of incoming migrants are smaller than counties closer to the coast. This can be explained by the relatively small populations and baseline levels of incoming migrants. The greater magnitudes of migrations from higher population areas causes *some* migrants to select these midwestern areas as destination, which could cause disproportionally larger *indirect* effects.

In general, we find that previously “unpopular” migrant destinations (areas with relatively low numbers of incoming migrants) would be more popular solely due to their close proximity to counties that experience “direct effects”. The East Coast will experience larger effects than the West coast because of the large coastal population centers and shallower coastlines, indeed, all counties adjacent to coastal counties on the East coast are marked as *indirectly* affected. Existing urban areas will receive the largest magnitudes of migrants, as they represent the most attractive destinations, which will accelerate the existing trends of urbanization. We find that the southeast portion of the United States will experience disproportionately high effects from SLR-driven flooding due to the large vulnerable populations in New Orleans and Miami. These results show that by driving human migration, the impacts of SLR have the potential to be much farther reaching than the coastal areas which they will flood.

Finally, we examine the effects that the choice of migration model has on our results in S1 Appendix. S1 and S2 Figs show results in the same format as Figs 2 and 3, however with an implementation of our general framework that uses the extended radiation model of human migration. Further discussion can be found in S1 Appendix.

## Discussion

The framework we propose here (Fig 1) introduces a systemic approach to couple models of climate change impacts with models of human migration. The framework has two improvements over our understanding about of the impacts of climate change on population by closely considering the corresponding effects on migration patterns. First, we posit that to capture the impacts of climate change, we need to consider how climate change affects population directly, “direct effects,” and indirectly through migration-driven heightened pressures on population centers, “indirect effects.” Second, we argue that calibrated models of climate impacts need to account for the fact that human responses to climate change, migration in our particular case, will differ from business as usual responses.

We apply our framework to analyze the impacts of sea level rise on human migration patterns and levels. We couple spatial estimates of SLR with human migration models and show that the effect of SLR on human populations could be more pervasive and widespread than anticipated, with almost all counties in the US receiving some number of additional migrants due to SLR induced flooding. Our results are the first step in understanding the socio-economic impacts of climate driven migration and more research is needed to understand how increased migration affects populations across different destinations.

While our framework is flexible enough in theory, in practice it will require assumptions specific to each application. These assumptions can be the result of knowledge gaps, data limitations, or large uncertainties in the individual component models. In our application to SLR, we have made several such assumptions that affect how we interpret the results of the coupled SLR/migration models. For example, we have assumed that people move due to SLR only when their homes are permanently flooded. It is of course possible, that people will move because their business or jobs are affected. According to the ‘Nuisance Hypothesis’ from Keenan et al. [40] housing prices are affected by people’s perceptions as to whether or not a property is at risk of flooding. This could impact “pull” factors of migration. While we cannot consider this channel in our current application due to data limitations, our framework allows to expand in this direction once more data is available.

Because we rely on machine learning techniques, we forfeit the explanatory power of our migration model in favor of a more accurate prediction. This limits our capacity to derive specific policy recommendations, but this approach is suitable for the purposes of our research question as it conceives a much more flexible methodology to analyze future migration. This black-box approach can be further calibrated as more data on similar temporal and spatial scales for empirical studies to explain migration behaviors becomes available [20].

The limitations of our application notwithstanding, we find some of our results are similar to previously published estimates of human migration under SLR in the USA. For example, like in Hauer 2017 [27], inland areas immediately adjacent to the coast, and urban areas in the southeast US will observe the largest effects from SLR driven migration. Our framework for modeling human migration, however, reveals several notable differences. For example, compared to Hauer 2017 [27], our results show more incoming migrants to Houston and Dallas—two larger cities closer to affected coastal areas. This result follows from our framing requirement that flows predicted under climate change do not necessarily follow historical flows. According to Hauer 2017, the Austin, Texas Core Based Statistical Area is expected to observe the largest effects out of all destinations, with over 800 thousand incoming migrants due to SLR. This result follows because Austin has consistently been one of the fastest growing US cities over the past decade, which is captured and projected by a time series based migration model. Our migration model instead captures the dynamics of human migration between US counties based on population and distance features, and uses this to predict flows between counties without regard to potential short term historic trends. Thus, our framework has the benefit of predicting flows between pairs of counties for which there are no historical flows. Our application also shows the “indirect impacts” could be important in magnitude. We observe that migrants from unaffected areas, that would previously move to coastal areas, will relocate to larger population centers. The counties surrounding Los Angeles in particular could see tens of thousands of migrants that are not coming from affected areas, but must choose a different location because their preferred coastal destination are now flooded.

While our application results are limited, our framework has shown conceptually how to think about widespread impacts of climate change. Moreover, as various aspects of human migration are better understood, especially ones related to environmental pressures, better models of human migration are created, and SLR flooding estimates are improved—with finer resolution population projections, uncertainty estimates, and models of the potential spatial effects of SLR such as expected flood frequency—we will be able to update our framework to produce more refined and comprehensive results.

## Supporting information

S1 Fig Extended radiation model spatial results. Map in the same format as Fig 2. but with extended radiation model results.
(TIF)

S2 Fig Extended radiation model indirect results. Map in the same format as Fig 3. but with extended radiation model results.
(TIF)

S3 Fig Ablation results for migration model. Maps showing the difference between results using separate models for climate change driven and “business as usual” migration and using the same model for both. Results shown for ANN and Extended radiation models.
(TIF)

S1 Table Comparison of migration model performance on different classes of migration. (XLSX)

S2 Table List of data sources. (XLSX)

S1 Appendix Sections on code and reproducibility, implementation and results with the extended radiation model, comparison of migration models, and ablation study on modeling climate migrants separately. (PDF)

## References

[1] Pachauri RK, Allen MR, Barros VR, Broome J, Cramer W, Christ R, et al. Climate change 2014: synthesis report. Contribution of Working Groups I, II and III to the fifth assessment report of the Intergovernmental Panel on Climate Change. IPCC; 2014.

[2] McLeman R, Smit B. Migration as an adaptation to climate change. Climatic change. 2006;76(1-2):31–53. 10.1007/s10584-005-9000-7

[3] Feng S, Krueger AB, Oppenheimer M. Linkages among climate change, crop yields and Mexico—US cross-border migration. Proceedings of the National Academy of Sciences. 2010;107(32):14257–14262. 10.1073/pnas.1002632107 PMC2922556 20660749

[4] Wilby RL, Keenan R. Adapting to flood risk under climate change. Progress in physical geography. 2012;36(3):348–378. 10.1177/0309133312438908

[5] Kahn ME. Climatopolis: how our cities will thrive in the hotter future. Basic Books (AZ); 2013.

[6] Borjas GJ, Monras J. The Labor Market Consequences of Refugee Supply Shocks. National Bureau of Economic Research; 2016.

[7] Gordon K. The Economic Risks of Climate Change in the United States. Risky Business; 2014.

[8] Shayegh S. Outward migration may alter population dynamics and income inequality. Nature Climate Change. 2017;7(11):828 10.1038/nclimate3420

[9] Luber G, McGeehin M. Climate change and extreme heat events. American journal of preventive medicine. 2008;35(5):429–435. 10.1016/j.amepre.2008.08.021 18929969

[10] Brown TC, Mahat V, Ramirez JA. Adaptation to future water shortages in the United States caused by population growth and climate change. Earth’s Future. 2019;7(3):219–234. 10.1029/2018EF001091

[11] Church JA, Clark PU, Cazenave A, Gregory JM, Jevrejeva S, Levermann A, et al. Sea Level Change (Chapter 13). Change 2013: The Physical Science Basis Contribution of Working Group I to the Fifth Assessment Report of the Intergovernmental Panel on Climate Change. 2013.

[12] Vermeer M, Rahmstorf S. Global sea level linked to global temperature. Proceedings of the National Academy of Sciences. 2009;106(51):21527–21532. 10.1073/pnas.0907765106 PMC2789754 19995972

[13] Sweet W, Kopp R, Weaver C, Obeyskera J, Horton R, Thieler E, et al. Global and regional sea level rise scenarios for the United States. NOAA/NOS Center for Operational Oceanographic Products and Services; 2017. NOAA Technical Report NOS CO-OPS 083.

[14] Güneralp B, Güneralp İ, Liu Y. Changing global patterns of urban exposure to flood and drought hazards. Global environmental change. 2015;31:217–225. 10.1016/j.gloenvcha.2015.01.002

[15] Crossett K, Ache B, Pacheco P, Haber K. National coastal population report, population trends from 1970 to 2020. National Oceanic and Atmospheric Administration. 2014;10.

[16] Hauer ME, Evans JM, Mishra DR. Millions projected to be at risk from sea-level rise in the continental United States. Nature Climate Change. 2016;6(7):691–695. 10.1038/nclimate2961

[17] Nicholls RJ. Analysis of global impacts of sea-level rise: a case study of flooding. Physics and Chemistry of the Earth, Parts A/B/C. 2002;27(32):1455–1466. 10.1016/S1474-7065(02)00090-6

[18] Willekens F, Massey D, Raymer J, Beauchemin C. International migration under the microscope. Science. 2016;352(6288):897–899. 10.1126/science.aaf6545 27199405 PMC5508757

[19] Black R, Adger WN, Arnell NW, Dercon S, Geddes A, Thomas D. The effect of environmental change on human migration. Global Environmental Change. 2011;21:S3–S11.

[20] Piguet E. Linking climate change, environmental degradation, and migration: a methodological overview. Wiley Interdisciplinary Reviews: Climate Change. 2010;1(4):517–524.

[21] Hugo G. Future demographic change and its interactions with migration and climate change. Global Environmental Change. 2011;21:S21–S33. 10.1016/j.gloenvcha.2011.09.008

[22] Wyett K. Escaping a rising tide: sea level rise and migration in Kiribati. Asia & the Pacific Policy Studies. 2014;1(1):171–185. 10.1002/app5.7

[23] Curtis KJ, Schneider A. Understanding the demographic implications of climate change: estimates of localized population predictions under future scenarios of sea-level rise. Population and Environment. 2011;33(1):28–54. 10.1007/s11111-011-0136-2

[24] Adamo SB, Sherbinin Ad. The Impact of Climate Change on the Spatial Distribution of Populations and Migration. In: Population distribution, urbanization, internal migration and development: An international perspective. United Nations; 2012. p. 161–195.

[25] Findlay AM. Migrant destinations in an era of environmental change. Global Environmental Change. 2011;21:S50–S58. 10.1016/j.gloenvcha.2011.09.004

[26] Thiede BC, Gray CL. Heterogeneous climate effects on human migration in Indonesia. Population and Environment. 2017;39(2):147–172. 10.1007/s11111-016-0265-8 31341345 PMC6656383

[27] Hauer ME. Migration induced by sea-level rise could reshape the US population landscape. Nature Climate Change. 2017 10.1038/nclimate3271

[28] Davis KF, Bhattachan A, D’Odorico P, Suweis S. A universal model for predicting human migration under climate change: examining future sea level rise in Bangladesh. Environmental Research Letters. 2018;13(6):064030 10.1088/1748-9326/aac4d4

[29] Marcy D, Brooks W, Draganov K, Hadley B, Haynes C, Herold N, et al. New mapping tool and techniques for visualizing sea level rise and coastal flooding impacts. In: Proceedings of the 2011 Solutions to Coastal Disasters Conference, Anchorage, Alaska, June 26 to June 29; 2011. p. 474.

[30] Robinson C, Dilkina B. A Machine Learning Approach to Modeling Human Migration. In: Proceedings of the 1st ACM SIGCAS Conference on Computing and Sustainable Societies. ACM; 2018. p. 30.

[31] Simini F, González MC, Maritan A, Barabási AL. A universal model for mobility and migration patterns. Nature. 2012;484(7392):96–100. 10.1038/nature10856 22367540

[32] Yang Y, Herrera C, Eagle N, González MC. Limits of predictability in commuting flows in the absence of data for calibration. Scientific reports. 2014;4. 10.1038/srep05662 PMC4092333 25012599

[33] Lenormand M, Huet S, Gargiulo F, Deffuant G. A universal model of commuting networks. PloS one. 2012;7(10):e45985 10.1371/journal.pone.0045985 23049691 PMC3462197

[34] Lenormand M, Bassolas A, Ramasco JJ. Systematic comparison of trip distribution laws and models. Journal of Transport Geography. 2016;51:158–169. 10.1016/j.jtrangeo.2015.12.008

[35] McLeman R. Developments in modelling of climate change-related migration. Climatic change. 2013; p. 1–13.

[36] U S Internal Revenue Service. Tax Stats—Migration Data; 2017. https://www.irs.gov/uac/soi-tax-stats-migration-data.

[37] Fussell E, Curtis KJ, DeWaard J. Recovery migration to the City of New Orleans after Hurricane Katrina: a migration systems approach. Population and environment. 2014;35(3):305–322. 10.1007/s11111-014-0204-5 24729651 PMC3979579

[38] O’Neill BC, Kriegler E, Riahi K, Ebi KL, Hallegatte S, Carter TR, et al A new scenario framework for climate change research: the concept of shared socioeconomic pathways. Climatic change. 2014;122(3):387–400. 10.1007/s10584-013-0905-2

[39] Erlander S, Stewart NF. The gravity model in transportation analysis: theory and extensions. vol. 3 Vsp; 1990.

[40] Keenan JM, Hill T, Gumber A. Climate gentrification: from theory to empiricism in Miami-Dade County, Florida. Environmental Research Letters. 2018;13(5):054001 10.1088/1748-9326/aabb32

