# Supplementary figures and images for: Modeling migration patterns in the USA under sea level rise
Source: PLoS One. 2020 Jan 22;15(1):e0227436. doi: 10.1371/journal.pone.0227436 (PMC6975524; doi:10.1371/journal.pone.0227436)

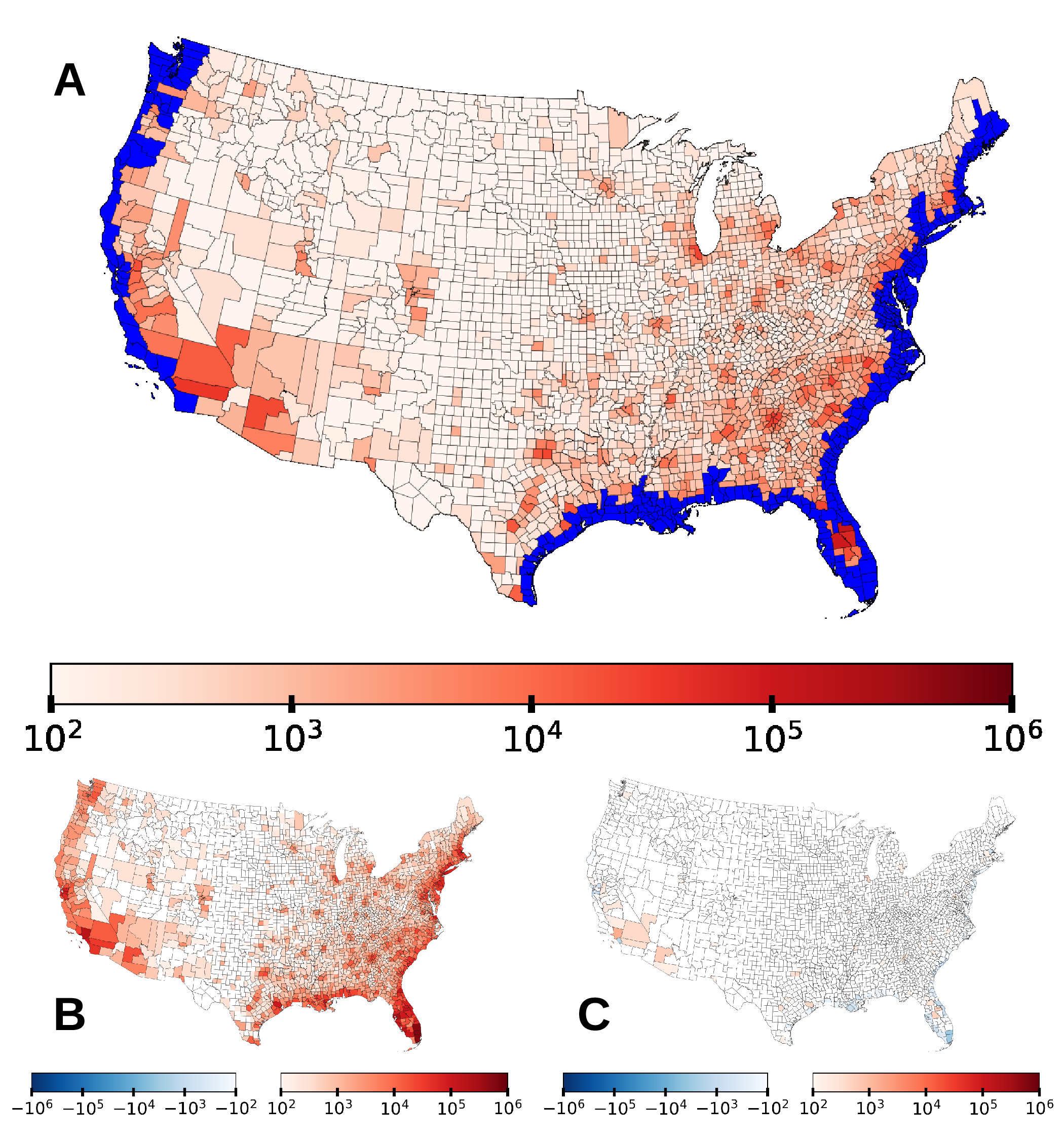

Supplement: S1 Fig — Map in the same format as Fig 2. but with extended radiation model results. (TIF) [file pone.0227436.s001.tif]

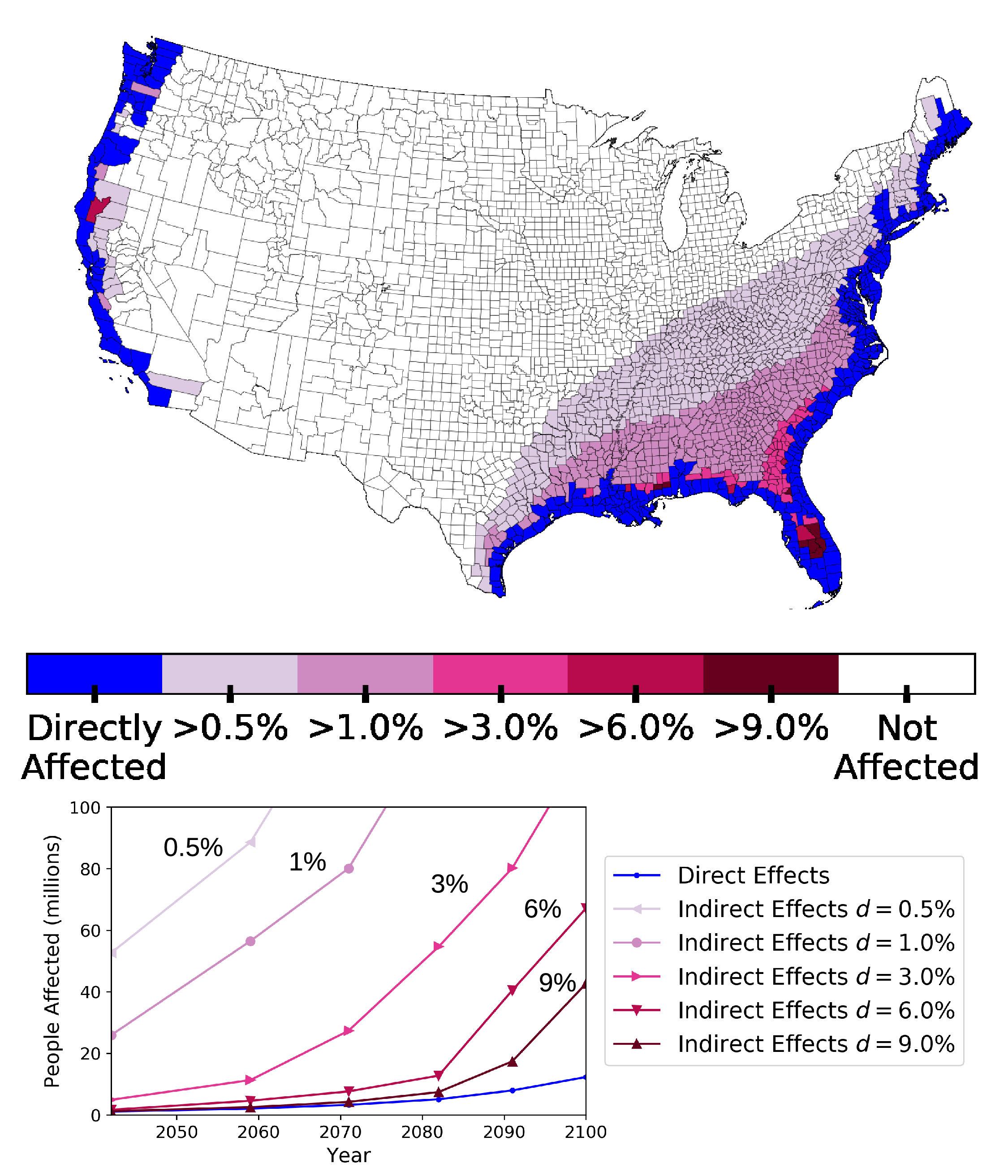

Supplement: S2 Fig — Map in the same format as Fig 3. but with extended radiation model results. (TIF) [file pone.0227436.s002.tif]

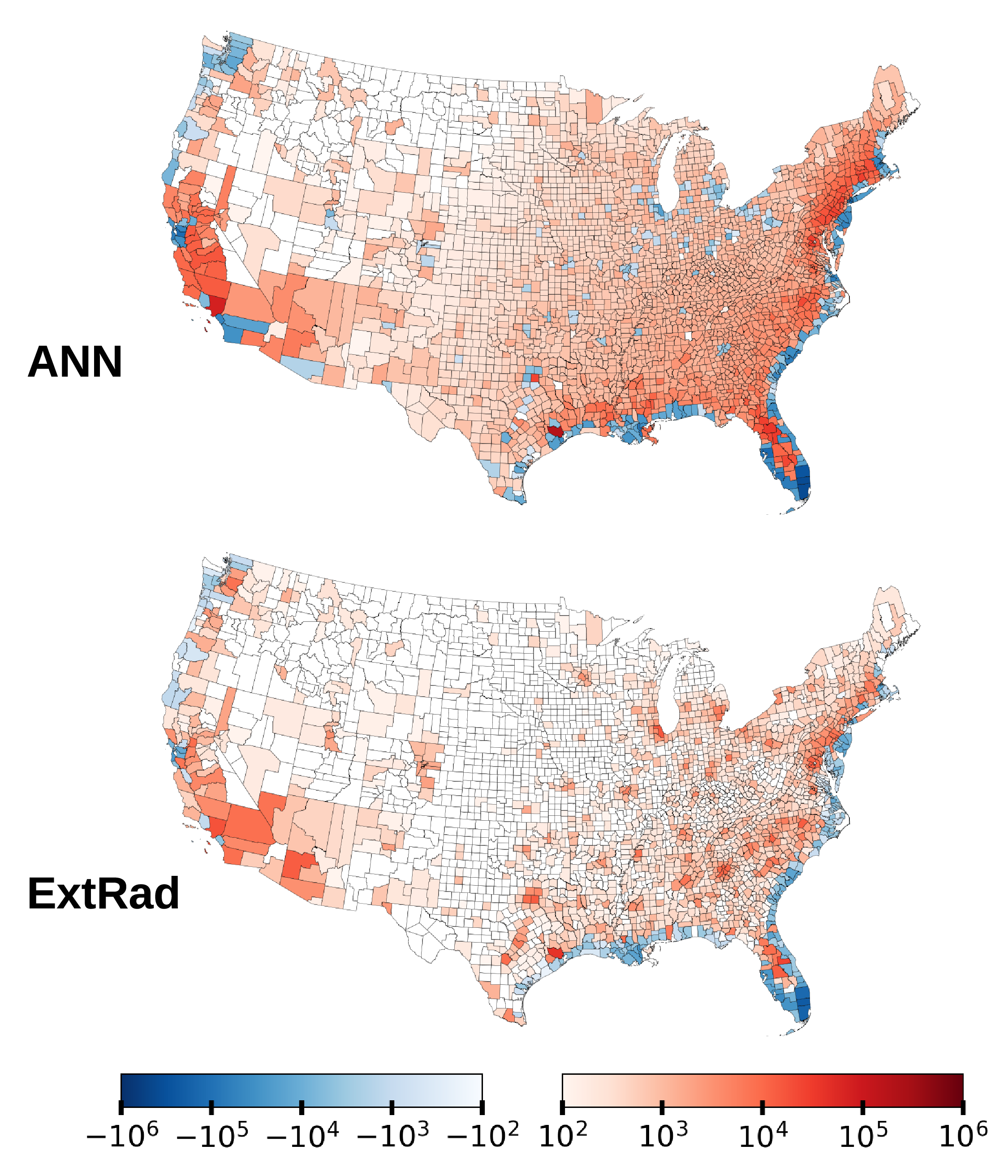

Supplement: S3 Fig — Maps showing the difference between results using separate models for climate change driven and “business as usual” migration and using the same model for both. Results shown for ANN and Extended radiation models. (TIF) [file pone.0227436.s003.tif]
