# Supplementary material for: Modeling migration patterns in the USA under sea level rise
Source: PLoS One. 2020 Jan 22;15(1):e0227436. doi: 10.1371/journal.pone.0227436 (PMC6975524; doi:10.1371/journal.pone.0227436)
Supplement: S1 Appendix — (PDF) [file pone.0227436.s006.pdf]

Supplementary Information - A modeling framework for studying migration patterns under climate change with an application to sea level rise

Caleb Robinson[1], Bistra Dilkina*[2], and Juan Moreno-Cruz[3]

[1]Georgia Institute of Technology, School of Computational Science and Engineering, Atlanta, Georgia, United States.
[2]University of Southern California, Viterbi School of Engineering, Los Angeles, California, United States.
[3]University of Waterloo, School of Environment, Enterprise and Development, Waterloo, Ontario, Canada.

August 25, 2019

1 Code and reproducibility

We have published open source code for reproducing the results in this paper at `https://github.com/calebrob6/migration-slr`. This effort includes scripts for processing the Digital Coast SLR data, reproducing the block group population estimates from Hauer et al. 2016 [1], training and running the artificial neural network (ANN) migration models described in Robinson and Dilkina [2], and creating all results and figures in the paper. We hope that this effort will encourage further study into the effects of sea level rise (SLR) on human migration and in other salient aspects of society.

1.1 Population projections

The population projections in this paper are reproduced from the methodology described in Hauer et al. 2016 [1]. This methodology involves modeling the growth (or decline) of housing units, h_i^t, for each census block group in the US based on historical data from 1940-2010, then projecting the number of housing units per census block group out to 2100. The population per housing unit, d_i, and the group quarters population, g_i, for each block group is assumed to stay constant at their 2010 values. Now, the total population for a particular block group, i, at some time, t, is given as $p_i^t = h_i^t * d_i + g_i$.

We find our estimates of affected county level populations in 2100 under both the medium and high SLR scenarios are similar to those reported in the the Supplementary Information of Hauer et al. 2016. These estimates can be downloaded in the accompanying code repository.

*Corresponding author: `dilkina@usc.edu`

2 Implementation and results using the Extended Radiation model

We implement our proposed framework using the extended radiation model [3] as a human migration model instead of the ANN model proposed in the main text. The extended radiation model is a rederivation of the original radiation model [4] under a survival analysis framework that includes the addition of a parameter which controls the influence of scale of the region and the degree of heterogeneity in the distribution of destination locations. Other human mobility models such as the gravity model [5, 6, 7, 8], and Schneider's intervening opportunities model [9] provide alternate ways to estimate the number of people that migrate between different locations.

The extended radiation model is given in Equation 1, where the variables are as follows: P_{ij} is the probability that a migrant who leaves zone i will travel to zone j, T_{ij} is the number of migrants that travel from zone i to j, m_i is the population of zone i, β is a parameter of the extended radiation model that controls the influence of scale of the region on trips, d_{ij} is the distance between zones i and j, and s_{ij} is the population in the circle centered at i with radius d_{ij} (which does not include m_i or m_j).

$$P_{ij} = \frac{[(m_i + m_j + s_{ij})^\beta - (m_i + s_{ij})^\beta](m_i^\beta + 1)}{[(m_i + s_{ij})^\beta + 1][(m_i + m_j + s_{ij})^\beta + 1]}, \quad i \neq j \tag{1}$$

As in the main text, we use two "versions" of the extended radiation model to model migrations from flooded areas and unflooded areas separately. We calculate β_C and β_S, the parameters for the models $MIGRATION_C$ and $MIGRATION_S$ respectively, by minimizing the *Common Part of Commuters* (CPC) metric [8] over the same sets of migration data that were used to train the previous ANN model. Here $\beta_C = 0.13$ and $\beta_S = 0.33$. We compare the fit of the ANN and Extended Radiation model in Section 3.

Figures 1 and 2 show the results from using these extended radiation models and are in the same format as the results figures from the main text. The general patterns match those from main text (which use an ANN model) - higher concentrations of migrants move to urban areas and counties immediately inland from coastal counties have higher percentages of incoming migrants by population. Figure 2 shows the severity of indirect effects degrading with distance from the East coast of the US, while showing that counties that are adjacent to the West coast will not experience similar effects. This result is a combination of the larger displaced populations along the East coast, and the relatively higher density of populations of counties in the Eastern US. The extended radiation model describes the probability of migration between two counties as decaying as a function of the intervening opportunities between them. The intervening opportunities between counties are fewer in the less dense counties of the Western US, meaning longer migrations will be more probable, while shorter migrations will be likely in the more dense Eastern US. Therefore, affected migrants leaving large populations centers along the east coast, such as Miami-Dade in Florida, will diffuse across the available opportunities at a rate roughly proportional to the distance from their origin - giving the observed pattern.

The results given by the extended radiation model predict larger amounts of indirect effects at the higher values of d than the ANN models do, i.e. that there will be larger numbers of people that are indirectly effected by SLR. This is a direct consequence of the diffusion pattern described in the previous paragraph, more population mass is dispersed over larger numbers of rural counties in the southeast US, thus counting the entire populations of these counties as being affected under higher values of d.

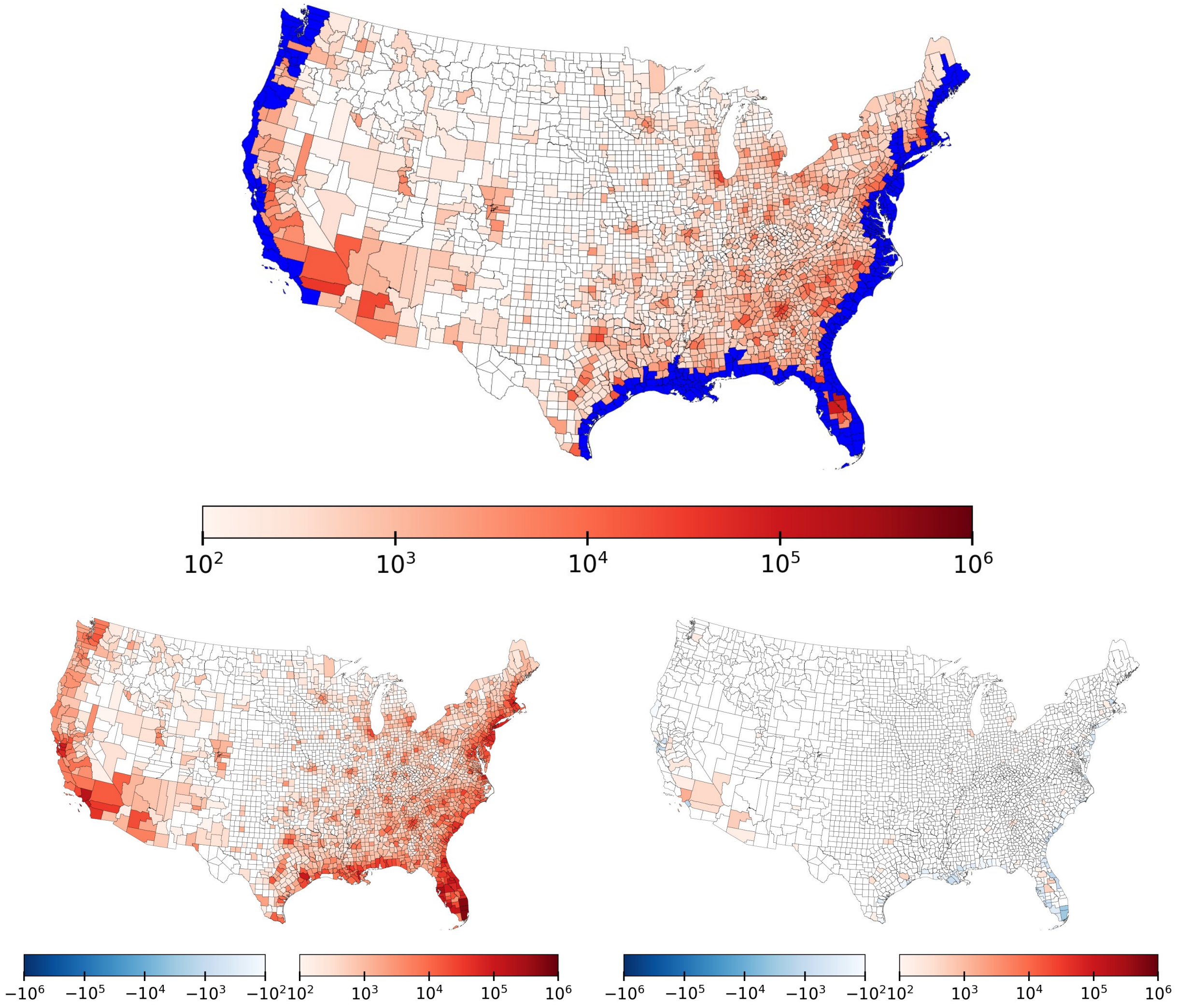

Figure 1: **Extended Radiation Model.** Spatial distribution of the direct and indirect effects of SLR on human migration. The **top** panel shows all counties that experience flooding under 1.8m of SLR by 2100 in blue and colors the remaining counties based on the number of additional incoming migrants per county that there are in the SLR scenario over the baseline. The **bottom left** map shows the number of additional incoming migrants per county in the SLR scenario from only flooded counties. The **bottom right** map shows the number of additional incoming migrants per county in the SLR scenario from only unflooded counties. Color gradients are implemented in a log scale.

3 Migration model training and validation

Our implementation of the Joint Model relies on ANN models of human migration to estimate migrations from affected and unaffected counties. We fit these models using historical county-to-county migration data from 2004-2014 from the IRS [10]. In this section, we evaluate the performance of our ANN models against the Extended Radiation [3], Radiation [4], and two types of Gravity models [7, 8]. We have three sets of counties: **all counties** which consists of every county from *all*

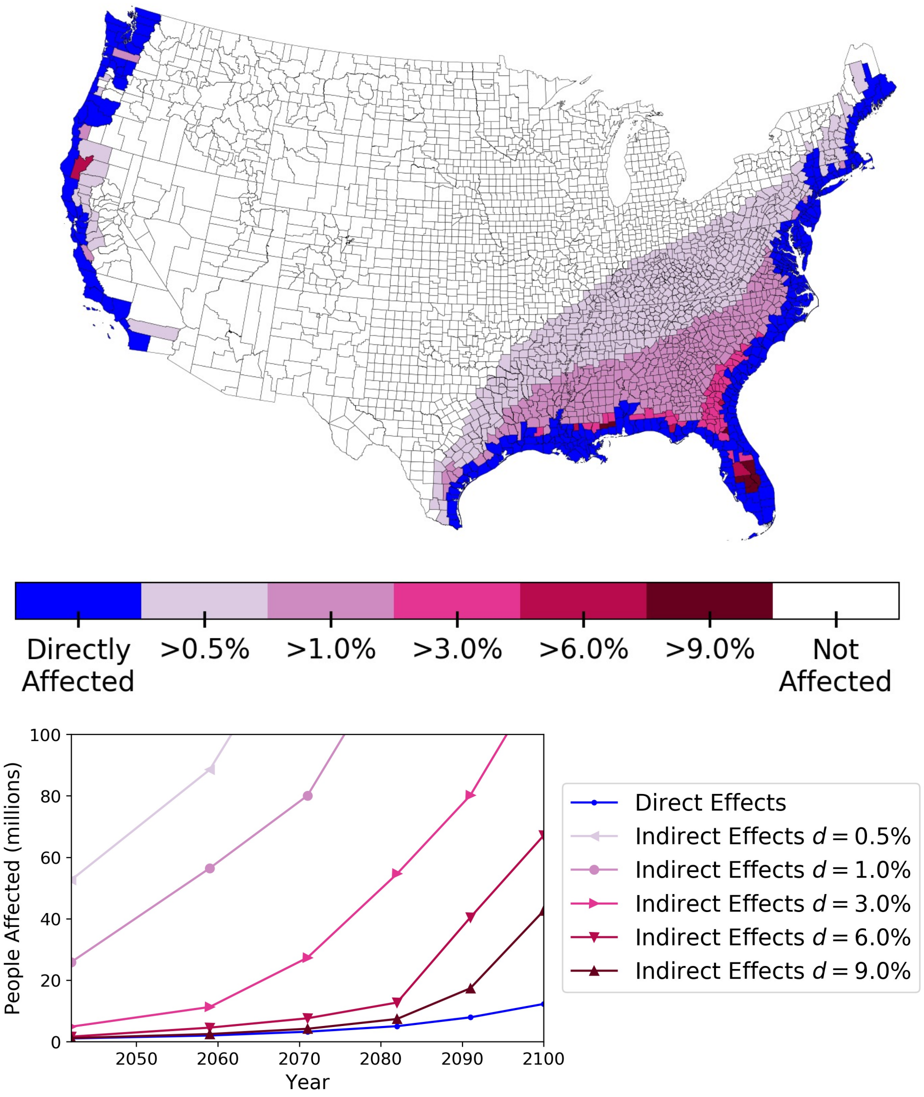

Figure 2: **Extended Radiation Model.** Impacts of SLR due to flooding and human migration for a range of SLR scenarios. In the **top** panel we show the spatial distribution of counties that are considered indirectly affected at different threshold values of d for the 1.8m SLR case in the southeast portion of the United States. In the **bottom** panel we show the number of people that are directly and indirectly affected under the same threshold values of d for the entire United States. We show the same impacts from Figure 3 in the main text.

years of migration data, **affected counties** which consist of the 7 counties most heavily impacted by Hurricanes Katrina and Rita in 2005 (discussed in the main text), and **unaffected counties** which consists of the set difference **all counties - affected counties**. We evaluate each model's average cross-validated performance in three tasks: estimating migrations from **all counties** to **all counties**, from **unaffected counties** to **unaffected counties**, and from **affected counties** to **unaffected counties**. In all of these cross-validation experiments we split on origin counties, i.e.

we select a set of training counties and use all migration observations originating in that set of counties (different rows of $\mathbf{T}$) to fit our models, then test on the migration observations originating in the remaining counties. In the **affected counties** to **unaffected counties** estimation task we use leave-one-out cross-validation as we only have 7 origin counties, while in the other two tasks we use 5-folds cross-validation. In each cross-validation fold we fit a model for every year of training data and average the results. This fitting procedure involves: training the ANN models using the parameters/architecture described in [2], estimating the single parameter, β, of the extended radiation or gravity models, and estimating the production function coefficient α. We estimate the β parameters by maximizing the CPC metric (described below) between the training migration data and modeled migration data. Finally, we estimate the α parameter by calculating the slope of the best fit line through all $(m_i, \sum_{j=1}^{n} T_{ij})$ points, i.e. computing the average fraction of the population of a county that migrates away in a given year. All parameter fitting is done only within a fold's training data, and the average and standard deviations of the α and best β parameters for each task is reported in Table 1.

To measure model performance we record the Common Part of Commuters (CPC) [7], Common Part of Commuters distance variant (CPC_d) [8], mean absolute error (MAE), and coefficient of determination (r^2) between the ground truth migrations, $\mathbf{T}$, and the model estimated migrations, $\hat{\mathbf{T}}$. These metrics are calculated on the origin destination migration matrices, hence we refer to them as "metrics on full matrix" in Table 1.

Common Part of Commuters (CPC) This metric directly compares numbers of travelers between the predicted and ground truth matrices. It will be 0 when the two matrices have no entries in common, and 1 when they are identical.

$$CPC(\mathbf{T}, \hat{\mathbf{T}}) = \frac{2\sum_{i,j=1}^{n} min(T_{ij}, \hat{T}_{ij})}{\sum_{i,j=1}^{n} T_{ij} + \sum_{i,j=1}^{n} \hat{T}_{ij}} \tag{2}$$

Common Part of Commuters Distance Variant (CPC_d) This metric measures how well a predicted migration matrix recreates trips at the same distances as the ground truth data. In this definition, N is a histogram where a bin N_k contains the number of migrants that travel between $2k-2$ and $2k$ kilometers. It will be 0 when the two matrices do not have any migrations at the same distance, and 1 when all fall within the same distances.

$$CPC_d(\mathbf{T}, \hat{\mathbf{T}}) = \frac{2\sum_{k=1}^{\infty} min(N_k, \hat{N}_k)}{\sum_{k=1}^{\infty} N_k + \sum_{k=1}^{\infty} \hat{N}_k} \tag{3}$$

Mean absolute error (MAE) This is a standard error measure, the average absolute difference between the predicted and ground truth values. Here, smaller values represent smaller errors in terms of number of migrants.

$$MAE(\mathbf{T}, \hat{\mathbf{T}}) = \frac{1}{n}\sum_{i,j=1}^{n}(T_{ij} - \hat{T}_{ij}) \tag{4}$$

Coefficient of determination (r^2) This score measures the goodness of fit between a set of predictions and the ground truth values. This score ranges from 1, in a perfect fit, to arbitrarily

negative values as a fit becomes worse, and is 0 when the predictions are equivalent to the expectation of the ground truth values.

$$r^2(\mathbf{T}, \hat{\mathbf{T}}) = 1 - \frac{\sum_{i,j=1}^{n}(T_{ij} - \hat{T}_{ij})^2}{\sum_{i,j=1}^{n}(T_{ij} - \bar{T})^2} \quad (5)$$

We also measure the aggregate model performance of predicting the aggregate incoming migrants per county. We calculate the MAE and r^2 between $T_{:i} = \sum_{j=1}^{n} T_{ji}$ and $\hat{T}_{:i} = \sum_{j=1}^{n} \hat{T}_{ji}$ and call them "metrics on incoming migrants vector" in Table 1.

The results of these experiments are shown in Table 1. Here, the ANN model outperforms the other models in the large **unaffected counties** to **unaffected counties** task and **all counties** to **all counties** tasks. In these two tasks the ANN model has a large amount of training data to exploit, and performs best in the matrix MAE, matrix r^2, and incoming migrants MAE metrics, and second best in the remaining metrics. The gravity model with power law decay performs best in terms of CPC and CPC_d on both tasks, however fails to appropriately model the pairwise flows with a matrix r^2 score of near 0. Similarly, the extended radiation model performs slightly better than the ANN model in terms of incoming migrants r^2 (and is indeed the only traditional migration model with a positive matrix r^2 metric), but performs significantly worse than the ANN model in all other dimensions.

In the **affected counties** to **unaffected counties** task, the best performing model is not obvious. Here, the matrix MAE and r^2 values are identical to the incoming migrants MAE and r^2 values due to the leave-one-out cross validation method - the test set in each split are all possible migrations originating from a single county. We observe that the extended radiation model performs the best in terms of CPC and r^2 scores, however with wildly different best parameter values between cross-validation splits (shown in the large standard deviation of the best β value). The ANN model is performing best in terms of CPC_d and MAE, but poorly on average considering the average r^2 score near 0. In the testing splits where the model is performing poorly it has overfit to the longer distance migrations observed in 6 out of the 7 **affected counties** and is unable to capture the shorter distance migrations from the held out county. As the extended radiation model captures the *general* behavior of human migration (with a single parameter), it does not overfit in this case. The best β parameters in the **affected counties** to **unaffected counties** task are significantly different than in the other two tasks, showing how the structure of migration may be different under extreme flooding events.

4 Effects of modeling climate migrants separately

In the main text we argue that persons living in areas that will be affected by SLR will be exposed to increased climatic pressures and will be forced to migrate elsewhere. Furthermore, we train an ANN model to separately model these migrations based on historic migration patterns from counties that were especially affected by Hurricanes Katrina and Rita. Here, we show the effect that separately modeling these migrations has on our results by simulating the same set of conditions with a single migration model for both types of migrants under both the ANN and Extended Radiation migration models. Figure 3 shows the difference in number of incoming migrants per county between the results using separate migration models and the results using a single migration model for the 1.8m SLR scenario. Notably, in both sets of results, when climate driven migrations are *not* modeled separately, then more migrations to coastal areas are predicted. One explanation for this pattern is that the

		Metrics on full matrix				Metrics on incoming migrants vector	
**Unaffected to Unaffected** Best $\alpha = 0.0325$ (0.0017)	Best β	CPC	CPC_d	MAE	R2	MAE	R2
Extended Radiation	0.3537 (0.0172)	0.4926 (0.0027)	0.7134 (0.0436)	1.0495 (0.3343)	0.1493 (0.1027)	287.7557 (84.2073)	**0.8588 (0.0328)**
Radiation	n/a	0.4327 (0.0109)	0.6426 (0.0611)	1.1816 (0.3992)	-0.6345 (0.1548)	326.2592 (89.5871)	0.7773 (0.0647)
Gravity Exponential Decay	0.2124 (0.3938)	0.3058 (0.1549)	0.3988 (0.2089)	1.4387 (0.5281)	-3.7014 (4.3919)	373.4170 (109.4751)	0.5119 (0.1897)
Gravity Power Law Decay	2.6955 (0.0829)	**0.5530 (0.0244)**	**0.8000 (0.0867)**	0.9421 (0.3589)	-0.0928 (0.8651)	341.2711 (128.2462)	0.5883 (0.1862)
ANN Model	n/a	0.5406 (0.0244)	0.7321 (0.0500)	**0.9272 (0.3413)**	**0.3687 (0.0587)**	**265.3275 (86.9390)**	0.8435 (0.0360)
**Affected to Unaffected** Best $\alpha = 0.1674$ (0.0326)	Best β	CPC	CPC_d	MAE	R2	MAE	R2
Extended Radiation	0.2403 (0.2478)	**0.4882 (0.0790)**	0.5149 (0.0820)	9.2474 (12.5452)	**0.5719 (0.1355)**	-	-
Radiation	n/a	0.4692 (0.1104)	0.4820 (0.1087)	11.7799 (16.6184)	-0.2258 (0.7936)	-	-
Gravity Exponential Decay	0.0047 (0.0002)	0.3889 (0.1446)	0.4184 (0.1544)	9.7246 (12.4281)	0.3068 (0.1964)	-	-
Gravity Power Law Decay	1.6250 (0.0772)	0.3742 (0.1288)	0.4038 (0.1414)	10.3996 (13.7485)	0.3081 (0.1584)	-	-
ANN Model	n/a	0.4231 (0.1275)	**0.5366 (0.2240)**	**9.0060 (10.0530)**	-0.0640 (0.5563)	-	-
**All to All** Best $\alpha = 0.0326$ (0.0017)	Best β	CPC	CPC_d	MAE	R2	MAE	R2
Extended Radiation	0.3544 (0.0169)	0.4923 (0.0031)	0.7145 (0.0436)	1.0551 (0.3254)	0.1467 (0.1012)	290.3686 (81.5585)	**0.8553 (0.0323)**
Radiation	n/a	0.4326 (0.0107)	0.6422 (0.0600)	1.1875 (0.3895)	-0.6341 (0.1644)	329.6186 (86.5392)	0.7718 (0.0656)
Gravity Exponential Decay	0.2124 (0.3938)	0.3062 (0.1551)	0.3997 (0.2091)	1.4451 (0.5209)	-3.7402 (4.4424)	376.9256 (107.9260)	0.5066 (0.1895)
Gravity Power Law Decay	2.6936 (0.0816)	**0.5522 (0.0240)**	**0.7997 (0.0863)**	0.9479 (0.3511)	-0.1035 (0.8609)	343.9989 (125.8610)	0.5822 (0.1836)
ANN Model	n/a	0.5445 (0.0237)	0.7367 (0.0556)	**0.9461 (0.3451)**	**0.3632 (0.0633)**	**275.1929 (90.9055)**	0.8357 (0.0440)

Table 1: Comparison of different models in predicting three different classes of migrations. Averaged cross-validation results are shown with standard deviations in parenthesis. In all columns except MAE, higher values represent better performance. Best performing number is bolded in each column, independently for each class of migration.

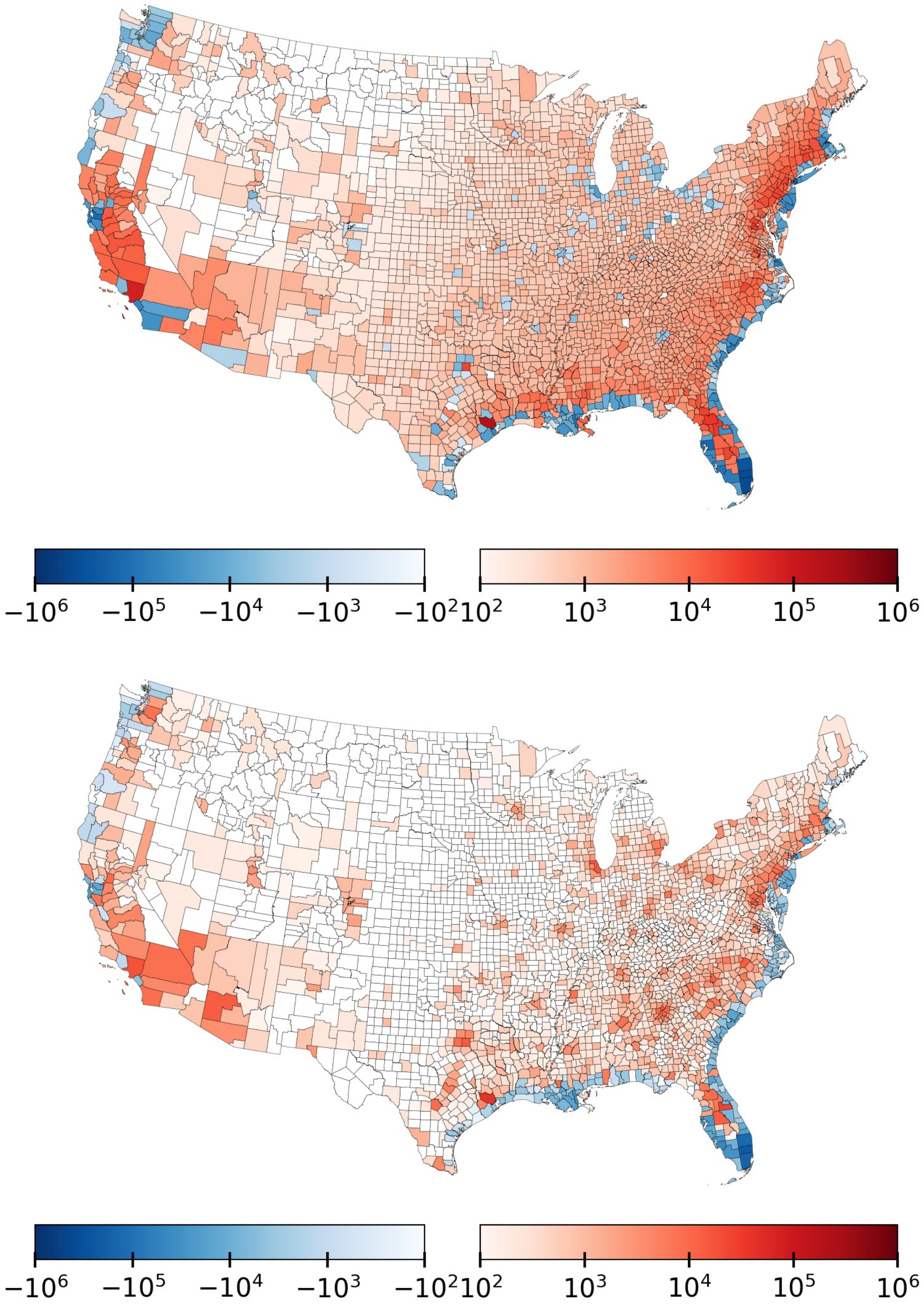

Figure 3: Difference in incoming migrants per county between migrations generated with separately trained models for affected and unaffected migrant behavior and migrations generated with a single model for both. The **top** panel shows ANN results, and the **bottom** panel shows Extended radiation model results. All results are for the High 2100 sea level rise/population scenario. In both sets of results the model that *does not* separately capture affected and unaffected migrant behavior predicts more incoming migrants to coastal counties.

probability of migration for climate driven migrations does not decay as strongly with distance as in standard migrations (e.g. the calibrated scale parameter in the Extended Radiation model is lower for climate migrants, which decreases the impact of intervening opportunities), therefore displaced migrants "see" distant population centers as attractive of a destination as nearby coastal destinations (which will usually be more populated than surrounding areas).

One discrepancy between the two sets of results is how, in the Extended radiation model results, separately modeled affected and unaffected migration behavior results in more predicted migrations to urban centers, while the corresponding ANN results show fewer predicted migrations to urban centers. This could also be due to the different emphasis on distance in predicting migration probabilities.

References

[1] M. E. Hauer, J. M. Evans, and D. R. Mishra, "Millions projected to be at risk from sea-level rise in the continental united states," *Nature Climate Change*, vol. 6, no. 7, pp. 691–695, 2016.

[2] C. Robinson and B. Dilkina, "A machine learning approach to modeling human migration," in *Proceedings of the 1st ACM SIGCAS Conference on Computing and Sustainable Societies*, p. 30, ACM, 2018.

[3] Y. Yang, C. Herrera, N. Eagle, and M. C. González, "Limits of predictability in commuting flows in the absence of data for calibration," *Scientific reports*, vol. 4, 2014.

[4] F. Simini, M. C. González, A. Maritan, and A.-L. Barabási, "A universal model for mobility and migration patterns," *Nature*, vol. 484, no. 7392, pp. 96–100, 2012.

[5] G. K. Zipf, "The P_1 P_2/D hypothesis: On the intercity movement of persons," *American Sociological Review*, vol. 11, no. 6, p. 677, 1946.

[6] S. Erlander and N. F. Stewart, *The gravity model in transportation analysis: theory and extensions*, vol. 3. Vsp, 1990.

[7] M. Lenormand, S. Huet, F. Gargiulo, and G. Deffuant, "A universal model of commuting networks," *PloS one*, vol. 7, no. 10, p. e45985, 2012.

[8] M. Lenormand, A. Bassolas, and J. J. Ramasco, "Systematic comparison of trip distribution laws and models," *Journal of Transport Geography*, vol. 51, pp. 158–169, 2016.

[9] M. Schneider, "Gravity models and trip distribution theory," *Papers in Regional Science*, vol. 5, no. 1, pp. 51–56, 1959.

[10] U.S. Internal Revenue Service, "Tax Stats - Migration Data." `https://www.irs.gov/uac/soi-tax-stats-migration-data`, 2017.
